# Overexpression of Lin28 Decreases the Chemosensitivity of Gastric Cancer Cells to Oxaliplatin, Paclitaxel, Doxorubicin, and Fluorouracil in Part via microRNA-107

**DOI:** 10.1371/journal.pone.0143716

**Published:** 2015-12-04

**Authors:** Rongyue Teng, Yan Hu, Jichun Zhou, Benjamin Seifer, Yongxia Chen, Jianguo Shen, Linbo Wang

**Affiliations:** 1 Department of Surgical Oncology, Sir Run Run Shaw Hospital, Hangzhou, Zhejiang Province, People’s Republic of China; 2 Zhejiang University, Hangzhou, Zhejiang province, People’s Republic of China; 3 Postgraduate Research Associate Yale School of Medicine, Department of Ob/Gyn & Reproductive Sciences, New Haven, Connecticut, United States of America; University of Kentucky College of Medicine, UNITED STATES

## Abstract

Higher Lin28 expression is associated with worse pathologic tumor responses in locally advanced gastric cancer patients undergoing neoadjuvant chemotherapy. However, the characteristics of Lin28 and its mechanism of action in chemotherapy resistance is still unclear. In this study, we found that transfection of Lin28 into gastric cancer cells (MKN45 and MKN28) increased their resistance to the chemo-drugs oxaliplatin (OXA), paclitaxel (PTX), doxorubicin (ADM), and fluorouracil (5-Fu) compared with gastric cancer cells transfected with a control vector. When knockdown Lin28 expression by Lin28 small interfering RNA (siRNA) was evaluated in vitro, we found that the resistance to chemo-drugs was reduced. Furthermore, we found that Lin28 up-regulates C-myc and P-gp and down-regulates Cylin D1. Finally, we found that miR-107 is a target microRNA of Lin28 and that it participates in the mechanism of chemotherapy resistance. Our results suggest that the Lin28/miR-107 pathway could be one of many signaling pathways regulated by Lin28 and associated with gastric cancer chemo-resistance.

## Introduction

Gastric cancer is a common clinical digestive system malignant tumor with a mortality rate that ranks third in the world [1]. Our country is an area that is prone to gastric cancer. In recent years, neo-adjuvant chemotherapy has been administered to gastric cancer patients to prolong survival. Nonetheless, the 5-year survival rate of those with advanced gastric cancer is still lower than 30% [2]. Chemotherapy resistance is the leading cause of the failure of gastric cancer and other tumor treatments. Therefore, the mechanism of chemotherapy resistance of gastric cancer is currently a major concern in gastric cancer studies.

Lin28 is a highly conserved RNA-binding protein that has been shown to participate in inducing pluripotent stem cells and in maintaining stem cell-like cells in cancer. Recent studies have reported that aberrant activation of Lin28 correlates well with the prognosis of different cancers, including breast, ovarian, lung, colon, and liver cancer [3–6]. Our previous study showed that Lin28 expression is also associated with poor survival in gastric cancer patients [7], and another study demonstrated that the expression of Lin28 is correlated with paclitaxel resistance in human breast cancer cells [8]. Moreover, Lin28 expression is associated with pathologic tumor response in locally advanced gastric cancer patients undergoing neo-adjuvant chemotherapy. However, the molecular mechanism underlying these findings remains unclear [9].

Lin28 promotes tumorigenesis by repressing tumor suppressor miRNAs such as the let-7 family, which is associated with drug resistance [8]. However, as a target miRNA of Lin28, miR-107 is rarely mentioned as a drug resistance factor in gastric cancer. MiR-107 expression is decreased in a variety of malignancies, such as colon cancer, head and neck cancer, pancreatic cancer and gastric cancer [10–13]. MiR-107 regulates downstream genes which are involved in cell differentiation and proliferation, metabolism, stress responses and the formation of blood vessels [10–14]. In this study, we investigated the Lin28/miR-107 pathway and its role in gastric cancer cell resistance to chemotherapy. We may have identified a new potential target for chemotherapy-resistant gastric cancer.

## Materials and Methods

### 1. Cell culture and plasmid transfection

The human gastric cancer cell lines MKN-28 and MKN-45 were obtained as a gift from Professor Sung Hoon Noh (Yonsei University College of Medicine, Seoul, Korea). The cells were cultured in RPMI 1640 medium (Sigma-Aldrich, St. Louis, MO, USA) supplemented with 10% fetal bovine serum (MP Biomedicals, Costa Mesa, CA, USA) in a humidified atmosphere containing 5% CO2 at 37°C. Oxaliplatin, paclitaxel, doxorubicin, and fluorouracil were purchased from Sigma (St. Louis, MO). The plasmid pcDNA3.1-Lin28 was transfected with Xtreme GENE HP DNA Transfection Reagent (Roche Applied Science, Mannheim, Germany) according to the manufacturer’s protocol. Stable Lin28-expressing clones were further selected and cultured with the appropriate neomycin-containing RPMI 1640 medium.

### 2. Real-time quantitative PCR

Real-time q-PCR analyses were performed with the ABI Prism 7500 sequence detection system (Applied Biosystems, Foster City, CA, USA). Briefly, a 20-μl reaction mixture containing 2 μl of cDNA template and 1 μl each of sense and anti-sense primers (Promega Gotaq q-PCR® Master Mix system, Madison, WI, USA) was amplified as follows: denaturation at 95°C for 10 min and 40 cycles of 95°C for 30 s, 60°C for 30 s, and 72°C for 40 s. Real-time q-PCR reactions were performed in triplicate for each sample, and the mean value was used to calculate mRNA levels. Quantitative analysis was performed using the comparative CT method. The Lin28 and miR-107 copy numbers in tumor tissues were normalized to the mRNA copy numbers of the housekeeping gene glyceraldehyde 3-phosphate dehydrogenase (GAPDH) to obtain the value 2 ^−Δ CT^. The primer sequences are as follows in Table 1.

**Table 1:** The primer sequences used for detecting drug resistant gene.

		primer
Lin28	forward	5’-AGCGCAGATCAAAAGGAGACA-3’
	reverse	5’-CCTCTCGAAAGTAGGTTGGCT-3’
GAPDH	forward	5’-CTTAGCACCCCTGGCCAAG-3’
	reverse	5’-GATGTTCTGGAGAGCCCCG-3’
Bcl-2	forward	5’-AGTGGGATGCGGGAGATGTG-3’
	reverse	5’-GGGATGCGGCTGGATGGG-3’
CDK6	forward	5’-GAGGTGGCGGTGCTGAGG-3’
	reverse	5’-GTTAGTTTGGTTTCTCTGTCTGTTCG-3’
NF-kB	forward	5’-AACAGCAGATGGCCCATACCT-3’
	reverse	5’-ACGCTGAGGTCCATCTCCTTG-3’
P-gp	forward	5’-GGTCACGCACAGCATG-3’
	reverse	5’-GTACACGGAAAGCTTGAC-3’
TOPOII	forward	5’-GCTGTGGATGACAACCTCCT-3’
	reverse	5’-GCTGTGGATGACAACCTCCT-3’
CyclinD1	forward	5’-CCCTCGGTGTCCTACTTCAA-3’
	reverse	5’-TGGCATTTTGGAGAGGAAGT-3’
C-myc	forward	5’-GCCTCAGAGTGCATCGAC-3’
	reverse	5’-TCCACAGAAACAACATCG-3’

### 3. Cell Drug-resistance Assay

Cell viability was measured using an MTT [3-(4,5-Dimethylthiazol-2-yl)-2,5-Diphenyltetrazolium Bromide] assay (Amresco, Solon, OH, USA) according to the manufacturer’s protocol. Cells were seeded in 96-well plates at a density of 5*10^3^ cells per well. After treatment, the cells were incubated with 5 mg/ml MTT for 4 h. Then, the medium was removed and 150 ml of sterilized DMSO solution was added, followed by incubation at 37°C for 4 h. The OD value was obtained at the wavelengths 570 nm and 630 nm. The OD value (570 nm to 630 nm) can indirectly reflect the cell number and activity according to the following formula: survival rate = dosed group OD570-630/no dose group OD570-630. A cell survival/drug concentration curve was then generated.

### 4. Western Blot Analysis

Cell lysates were separated by electrophoresis on a 4–15% sodium dodecyl sulphate-polyacrylamide gradient minigel (SDSPAGE) (Bio-Rad, Hercules, CA) and electrophoretically transferred to a nitrocellulose membrane (Amersham Pharmacia, Piscataway, NJ). Western blots were probed with antibodies against Lin28, GAPDH (Santa Cruz Biotechnology, Santa Cruz, CA), caspases and cleaved caspases, Cyclin D1, NF-kB (Cell Signaling, Beverly, MA), CDK6, C-myc (Abcam Trading (Shanghai) Company, Shanghai, China), P-gp, and Bcl-2 (Huan Biotechnology, Beijing, China). The protein bands were detected by enhanced chemiluminescence (Amersham Pharmacia, Piscataway, NJ).

### 5. Flow Cytometric Assay

Cells (2*10^5^ per well) were plated in a six-well plate and treated with paclitaxel. After 72 h, cells were fixed in 70% ethanol and stained with propidium iodide (PI). The DNA content was analyzed with an Epics Profile II flow cytometer (Beckman Coulter, Fullerton, CA) with Multicycle software (Phoenix Flow Systems, San Diego, CA). All the experiments were repeated at least twice. PI was also used in conjunction with Annexin V (BD Biosciences, San Jose, CA) to determine if cells were viable, apoptotic, or necrotic.

### 6. MiRNA Transfection and Detection

Cells were plated in six-well culture plates and transfected with 50 nM pre-miR-107 purchased from Applied Biosystems (Carlsbad, CA) or control pre-miRNA according to the manufacturer’s protocol. Quantitative Real-time Polymerase Chain Reaction (PCR) was performed using the TaqMan MicroRNA Reverse Transcription Kit and the Fast Real-Time PCR System (Applied Biosystems, Carlsbad, CA) according to the manufacturer’s protocols. The fold change in miR-107 microRNA levels was calculated and normalized to a hsa-mir-423 loading control.

### 7. Animals

BALB/c mice aged 3–4 weeks weighing 18–22 g were purchased from SLAC laboratory animal company (SCXK-2007-004, Shanghai, China) and housed five per cage in a specific pathogen-free (SPF) environment. The animals were allowed to acclimate to the housing facilities for 7 days before the experiments began. Animal handling procedures were performed in accordance with the P. R. China legislation on the use and care of laboratory animals and approved by the Experimental Animal Ethical Committee of Zhejiang University (Zhejiang, China).

### 8. Xenograft Assay and Treatment

Briefly, groups of six BALB/c mice were injected subcutaneously with 1*10^6^ MKN45/Lin28, MKN45/Vector, or MKN45 cells in the right flank. The tumor volume was calculated using the formula: V = 1/2*a*b ^2^, where *a* and *b* are the longest and the shortest diameters of the tumor mass (in millimeters), respectively. When the tumors reached approximately 80 mm^3^, the mice were treated with injection of ADM (50mg/kg) and OXA (5uM/kg) every week for 2 weeks. Quantitative changes in body weight and tumor volume were measured every three days by a single individual using a balance and Vernier calipers. No anesthetics were used in the mouse experiments.

### 9. Statistical analysis

All the experiments were performed three times with triplicate samples. Analysis of variance and Student’s t-test were used to compare the values of the test and control samples. A P-value less than 0.05 was deemed to be statistically significant. Statistica 6.1 software was used for all statistical analyses. The significance was evaluated by the student t-test.

## Results

### 1. In vitro testing of chemotherapy sensitivity changes in gastric cancer cells stably expressing Lin28

**Fig 1:**
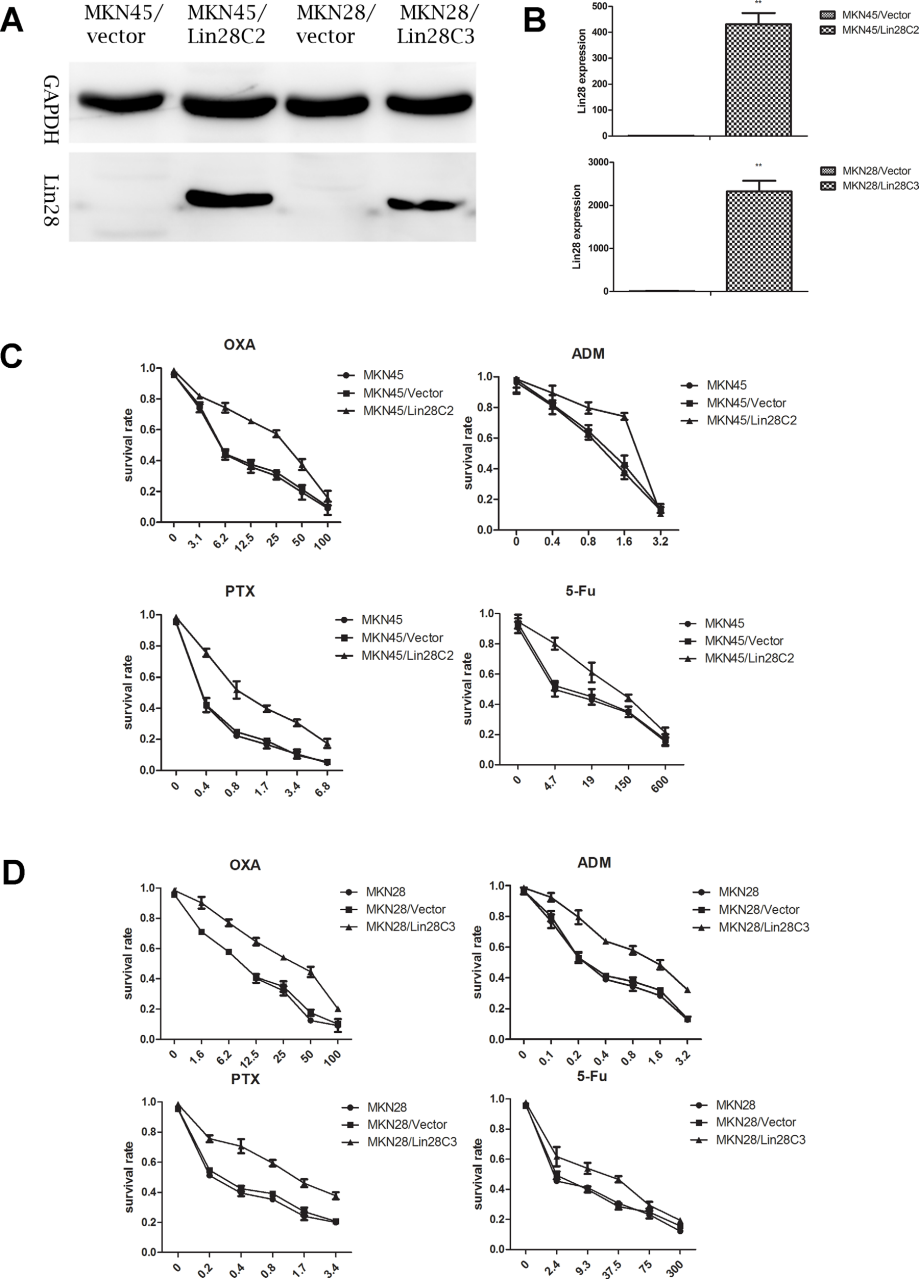
Lin28 increase chemotherapeutic drug resistance in gastric cancer. (A). Gastric cancer cell line MKN45 and MKN 28 with Lin28 overexpression were established via Lin28 plasmid transfection, Lin28 expression was detected by Western-blot.(B). mRNA level of Lin28 was detected by qRT-PCR, each data point represents the mean±SD of three independent experiments. (**p< 0.01). (C). MKN45 with Lin28 overexpression showed increased resistance to OXA, PTX, ADM, 5-Fu. (D). MKN28 with Lin28 overexpression showed increased resistance to OXA, PTX, ADM, 5-Fu.

Two strains of gastric cancer cells with negative Lin28 expression (MKN45 and MKN28) were selected and cultured. We identified gastric cancer cells with stable Lin28 expression from resistant transfected clones (MKN45/Lin28/C2 and MKN28/Lin28/C3) using western blotting and q-PCR (Fig 1).

The selected MKN45/Lin28C2, MKN45/Vector, MKN45, MKN28/Lin28C3, MKN28/Vector, and MKN28 cells were tested for chemotherapy drug susceptibility. We selected the following four commonly used clinical chemotherapy drugs for treatment of gastric cancer: OXA, PTX, ADM, and 5-Fu. Treatment with OXA, PTX, and ADM lasted for 48 hours, while 5-Fu treatment lasted for 72 hours. After Lin28 transfection, MKN45 and MKN28 cells had a decreased sensitivity to OXA, PTX, ADM, and 5-Fu that was most pronounced for OXA and PTX. The IC50 values were more than twice that of the control group (Fig 1).

**Fig 2:**
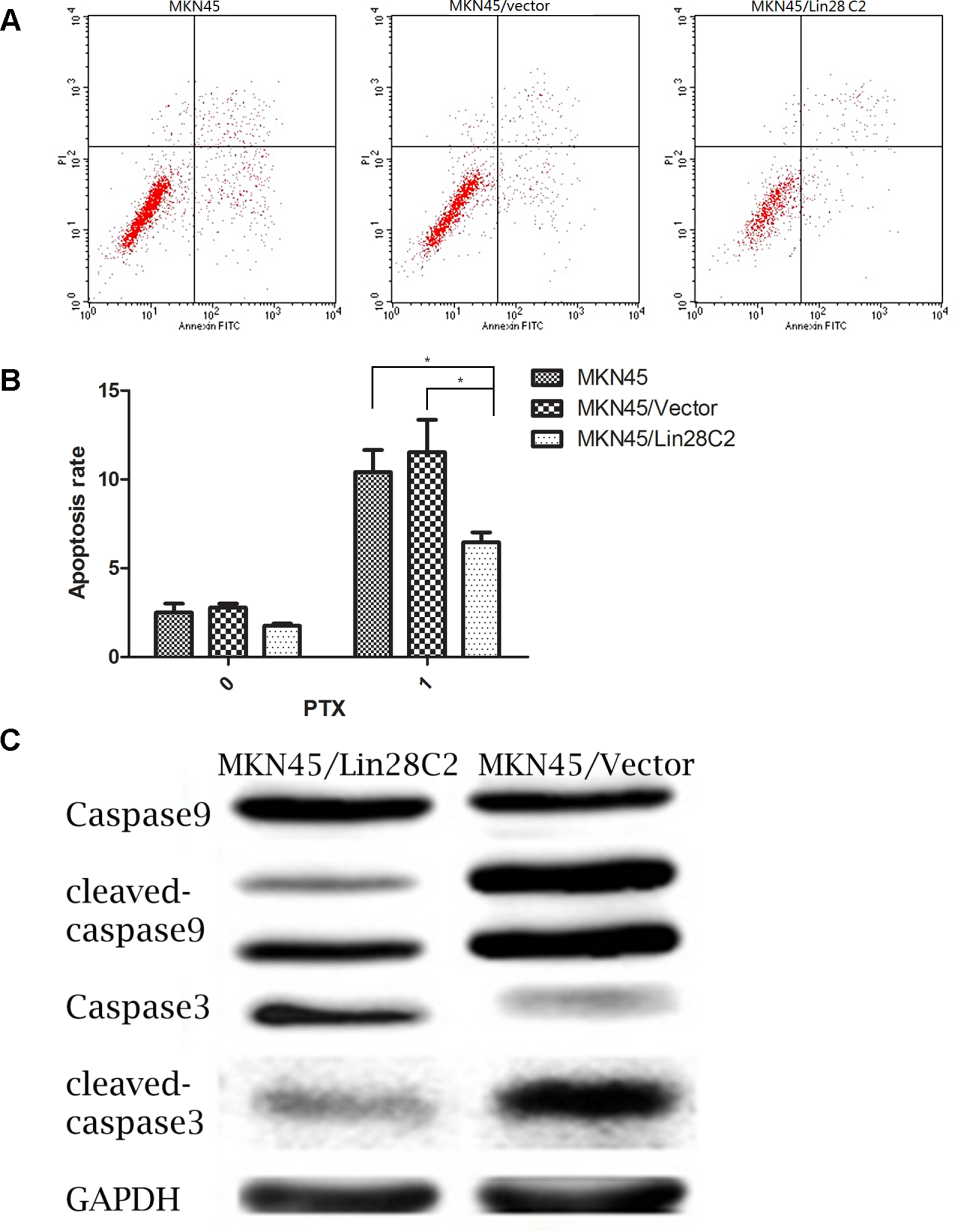
Lin28 suppressed apoptosis rate of MKN45 cells treated with PTX. (A) and (B). Apoptosis rate of MKN45/Lin28、MKN45/Vector、MKN45 treated with PTX at 0.1uM was displayed. Data showed MKN45/Lin28 had less apoptosis rate than MKN45/Vector and MKN45. Each data point represents the mean±SD of three independent experiments. (*p<0.05). (C). Cleaved- caspase9 and caspase3 were detected by Western blot. Cleaved- casepase 9 and casepase 3 were decreased in MKN45/Lin28.

On the basis of the MTT assay, we used flow cytometry to detect the differences in apoptosis between Lin28-positive and Lin28-negative cells after chemotherapy PTX exposure. The MKN45/Lin28, MKN45/Vector and MKN45 cell groups received 0 μM and 0.1 μM PTX treatment for 24 hours, and double staining was performed to detect cell apoptosis. The apoptotic rate of transfected Lin28 cells was lower than that of non-transfected cells (p<0.05). In addition, western blots of protein collected from cells treated with 0.1 μM PTX showed that apoptosis was reduced in MKN45/Lin28 cells as shown by decreases in the activation of caspase 9 and caspase 3 (Fig 2).

**Fig 3:**
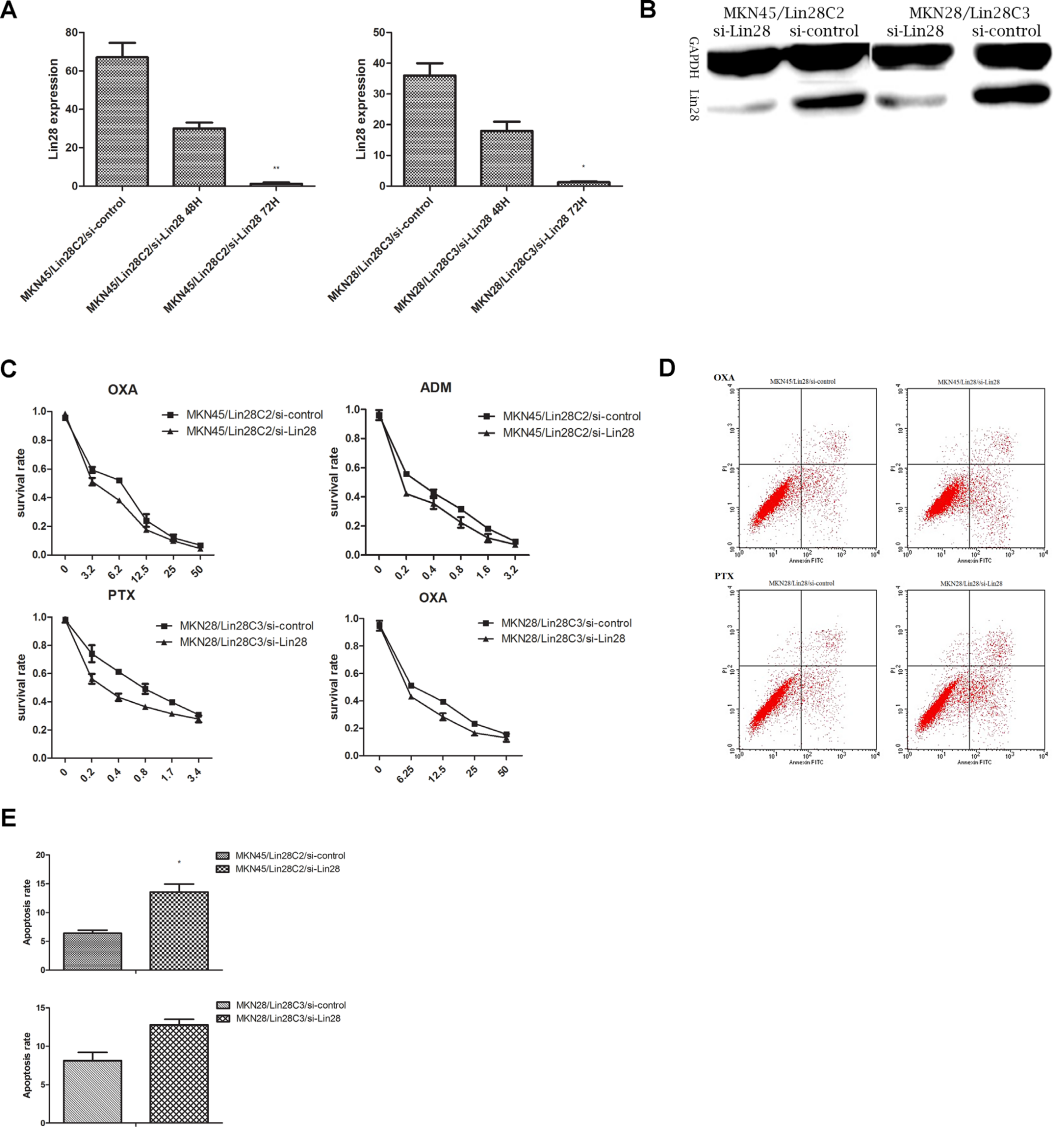
Knockdown Lin28 via siRNA increased chemotherapeutic sensitivity. (A).MRNA level of Lin28 after Lin28 knockdown were detected by qRT-PCR at 48h,72h post-transfection in MKN45/Lin28 C2 and MKN28/Lin28 C3. Each data point represents the mean±SD of three independent experiments. (**p<0.01,*p<0.05).(B). Protein level of Lin28 in MKN45/Lin28 C2 and MKN28/Lin28 C3 were detected by western-blot 72h after Lin28-siRNA transfection. (C). Chemo-sensitivity of MKN45/Lin28 and MKN28/Lin28 to OXA, PTX and ADM by MTT assay after Lin28 expression were knockdown. (D) and (E).Apoptosis change among MKN45/Lin28/si-Lin28, MKN45/Lin28/si-control, MKN28/Lin28/si-Lin28, MKN28/Lin28/si-control at OXA 3.2ug/ml,PTX 1uM.After Lin28 was knock-down,the apoptosis of MKN45/Lin28 were decreased. Each data point represents the mean±SD of three independent experiments. (*p<0.05).

Transient transfection of siRNA-Lin28 should inhibit Lin28 expression levels in MKN45/Lin28 and MKN28/Lin28 cells 48 hours after transfer according to the experimental guidelines of the reagent manufacturer. Lin28 protein expression was detected, and the associated drug sensitivity was tested. Lin28 RNA expression in gastric cancer cells was detected by q-PCR, while protein expression was detected by western blotting (Fig 3).

Q-PCR showed that Lin28 mRNA expression in MKN45/Lin28/si-Lin28 (48 h) and MKN45/Lin28/si-Lin28 (72 h) cells was much lower than that in MKN45/Lin28/si-control cells (p<0.01). This transfection effect was also verified in MKN28/Lin28 cells. Western blotting revealed that Lin28 si-RNA significantly inhibited Lin28 protein expression in MKN45/Lin28 and MKN28/Lin28 cells.

An MTT assay was performed on the four cell types (MKN45/Lin28/si-control, MKN45/Lin28/si-Lin28, MKN28/Lin28/si-control, and MKN28/Lin28/si-Lin28 cells) that were transfected with Lin28 siRNA for 48 hours to assess chemotherapy sensitivity. We chose to use the chemotherapy drugs that had the most pronounced effects in the pre-testing: OXA, PTX and ADM. We selected a concentration gradient of 5–6 according to the preliminary results of preliminary experiments. Interference by siRNA reduced the expression of Lin28, and Lin28-positive cell lines had an increased sensitivity to chemotherapeutic drugs (Fig 3).

We next detected the sensitivity of cells to different chemotherapy drugs after down-regulation of Lin28. Cells were divided into 4 groups, MKN45/Lin28/si-control, MKN45/Lin28/si-Lin28, MKN28/Lin28/si-control and MKN28/Lin28/si-Lin28, and were tested with the chemotherapy drugs OXA and PTX respectively. The chemotherapy drug concentrations (OXA 3.2 μg/mL and PTX 1 μM) were selected according to the results of preliminary experiments. The number of apoptotic MKN45/Lin28 and MKN28/Lin28 cells was less than that of MKN45/Lin28/si-Lin28 and MKN28/Lin28/si-Lin28 cells (p<0.05). This result indicates that down-regulation of Lin28 expression can increase cell apoptosis (Fig 3).

### 2. The molecular mechanisms by which Lin28 changes in chemotherapy sensitivity

**Fig 4:**
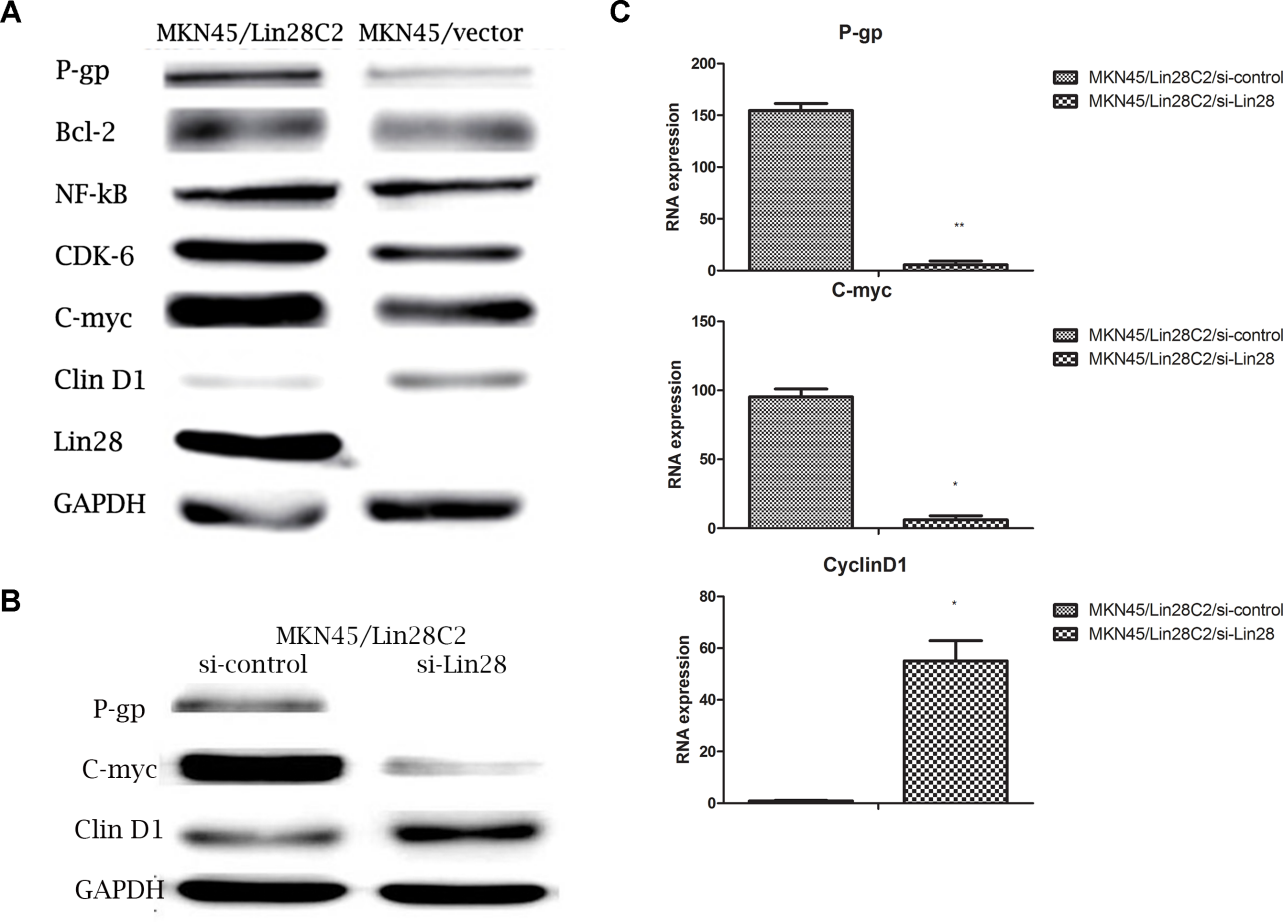
Lin28 influence the expression level of drug resistance-associated protein. (A).Differential expression of drug resistance-associated protein between MKN45/Lin28 and MKN45/Vector. (B)and(C).P-gp and C-myc expression level were decreased in both protein and RNA levels after Lin28 knockdown in MKN45/Lin28 cell, while Cyclin D1 expression was increased. Each data point represents the mean±SD of three independent experiments. (**p<0.01,*p<0.05).

We detected drug resistance-related gene expression levels by q-PCR in MKN45/Lin28 and MKN45/vector cells.P-gp, and C-myc RNA levels were markedly increased and Cyclin D1 RNA level were decreased, while other Bcl-2,CDK6,NF-kB,TOP II did not show significant difference between MKN45/Lin28 and MKN45/vector cells.Also these drug resistance-related gene expressions were investigated by western blot in MKN45/Lin28 and MKN45/Vector cells, which showed consistent trend that P-gp and C-myc protein expression increased while Cyclin D1 decreased. However, Bcl-2 expression decreased, NF-kB expression did not change, and expression of Cyclin D1 and the protein kinase CDK6 was reduced (Fig 4).

After Lin28 expression was inhibited by transfection with Lin28 siRNA, the RNA levels of P-gp, C-myc and Cyclin D1 were reassessed. After reduction in the RNA level of Lin28, P-gp and C-myc were significantly down-regulated, while Cyclin D1 levels significantly increased. Detection of P-gp, C-myc, and Cyclin D1 expression by western blotting revealed that after inhibition of Lin28 expression, P-gp and C-myc protein expression was significantly down-regulated, while the expression of Cyclin D1 was significantly upregulated (Fig 4).

### 3. MiR-107 is the target microRNA of Lin28

**Fig 5:**
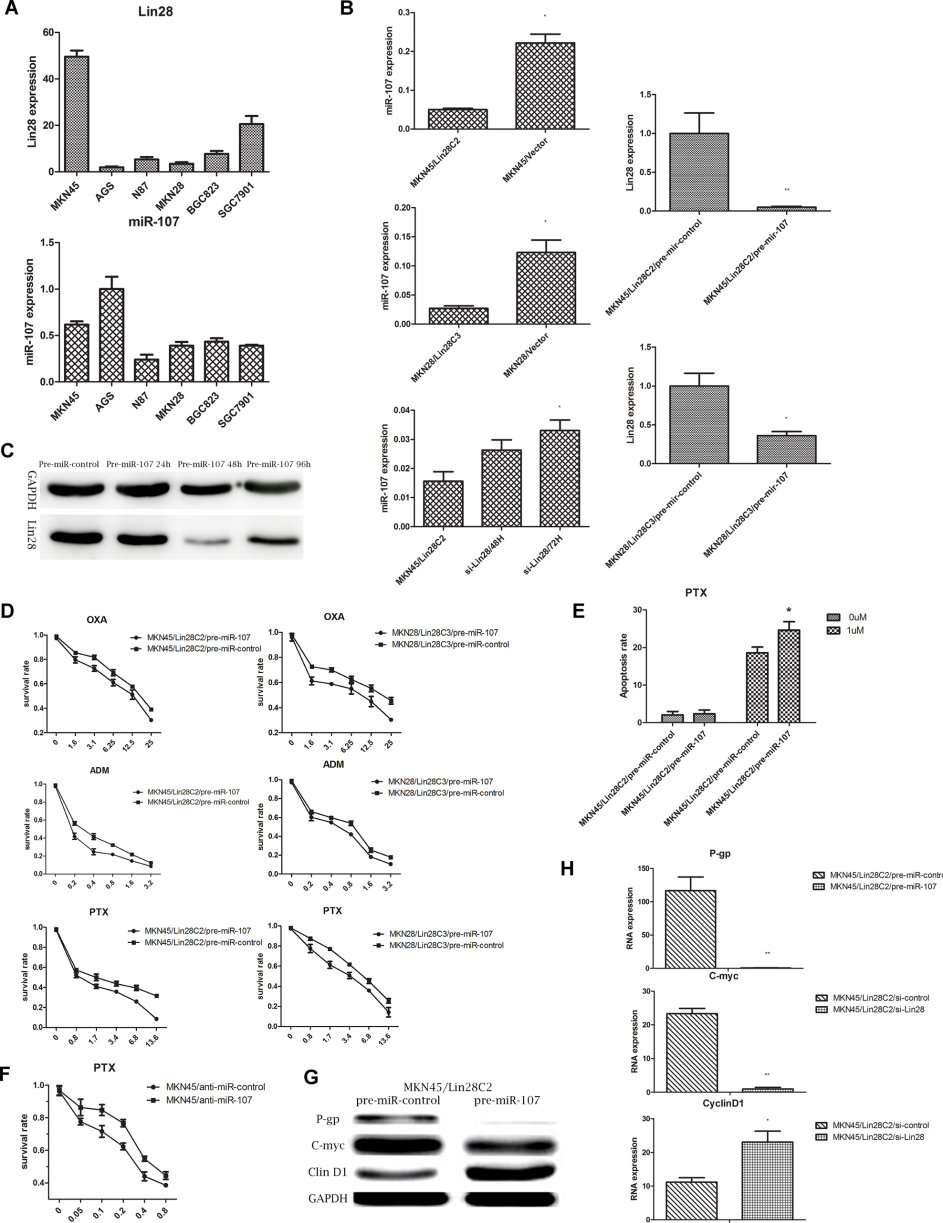
MiR-107 was involved in Lin28-mediated chemo-drug resistance. (A).RNA level of Lin28 and miR-107 were detected by qRT-PCR in gastric cell line. (B).MiR-107 expression level were detected by qRT-PCR after Lin28 overexpression (in MKN45 and MKN28) or Lin28 knockdown (in MKN45/Lin28). (C). Pre-miR-107 transfection down-regulated Lin28 in MKN45/Lin28 in a time-dependent manner. (D). Chemo-drug sensitivity of MKN45/Lin28C2 and MKN28/Lin28C3 increased after transfection of pre-miR-107. (E). Apoptosis rate of MKN45/Lin28 (treated with OXA at 3.2ug/ml) increased after transfected with pre-miR-107. (F). Chemo-drug sensitivity of MKN45 cell changed after transfected anti-miR-107. (G) and (H). P-gp, C-myc expression were decreased, while CyclinD1 expression was increased in MKN45/Lin28 cells after transfected with pre-miR-107.Each data point represents the mean±SD of three independent experiments. (**p<0.01,*p<0.05).

While testing the Lin28 RNA and miR-107 levels in gastric cancer cell lines, we found mutual inhibition of Lin28 and miR-107 in the MKN45 and AGS cell lines. Additionally, miR-107 was down-regulated in MKN45/Lin28 and MKN28/Lin28 cells. Knockdown of the expression of Lin28 reversed this inhibition. In a follow-up experiment, Lin28-positive cells, MKN45/Lin28 and MKN28/Lin28, were transfected with pre-mir-107 and were subsequently referred to as MKN45/Lin28/pre-miR-107 and MKN28/Lin28/pre-miR-107 cells. Transfected pre-miR-control cells were called MKN45/Lin28/pre-miR-Control and MKN28/Lin28/pre-miR-Control. Anti-miR-107 or control transfections into Lin28-negative cells, MKN45/Vector, yielded MKN45/Vector/anti-miR-107 and MKN45/Vector/anti-miR-Control cells, respectively. Forty-eight hours after transfection, RNA was collected and subjected to q-PCR to detect the differences in miR-107 expression in MKN45/Lin28, MKN28/Lin28, MKN45/Vector, and MKN28/Vector cells. The effect of knockdown of Lin28 expression by Lin28 siRNA was also assessed. After transfection with Lin28, miR-107 expression was significantly down-regulated, while after knockdown of Lin28 expression, the expression of miR-107 increased. Forty-eight hours after transfection of pre-miR-107, Lin28 RNA levels were significantly decreased, and 96 hours after transfection, Lin28 protein expression was still lower than that of the non-transfected control group. This finding indicates that miR-107 may regulate the expression of Lin28 (Fig 5).

### 4. MiR-107 participates in Lin28-mediated chemo-drug resistance

The chemotherapy sensitivity of 4 cell groups, MKN45/Lin28/pre-miR-control, MKN45/Lin28/pre-miR-107, MKN28/Lin28/pre-miR-control and MKN28/Lin28/pre-miR-107, was tested using the MTT method 48 hours after transfection with pre-miR-107. We chose chemotherapy drugs that had the most pronounced effects during pre-testing, OXA, PTX and ADM, and selected a concentration gradient of 5–6 according to preliminary results from preliminary experiments. The results suggest that the introduction of pre-miR-107 into Lin28-positive cells can increase cell sensitivity to chemotherapy. Apoptosis testing showed that the number of MKN45/Lin28 apoptotic cells increased after transfection with pre-miR-107 (p<0.05). MTT testing of MKN45/Lin28/anti-miR-control and MKN45/Lin28/anti-miR-107 chemotherapy sensitivity showed that inhibition of expression of miR-107 in MKN45 reduced the sensitivity to PTX (Fig 5).

By western blotting and q-PCR, we detected the drug resistance-related gene and protein expression levels in MKN45/Lin28 cells that had been transfected with pre-miR-107, including those of C-myc, P-gp, and Cyclin D1. The results showed that 48 hours after transfection with miR-107, the RNA and protein expression of C-myc and P-gp decreased (p<0.01), while the expression of Cyclin D1 increased (p<0.05) (Fig 5).

### 5. Lin28 associated chemo-drug resistance in vivo

**Fig 6:**
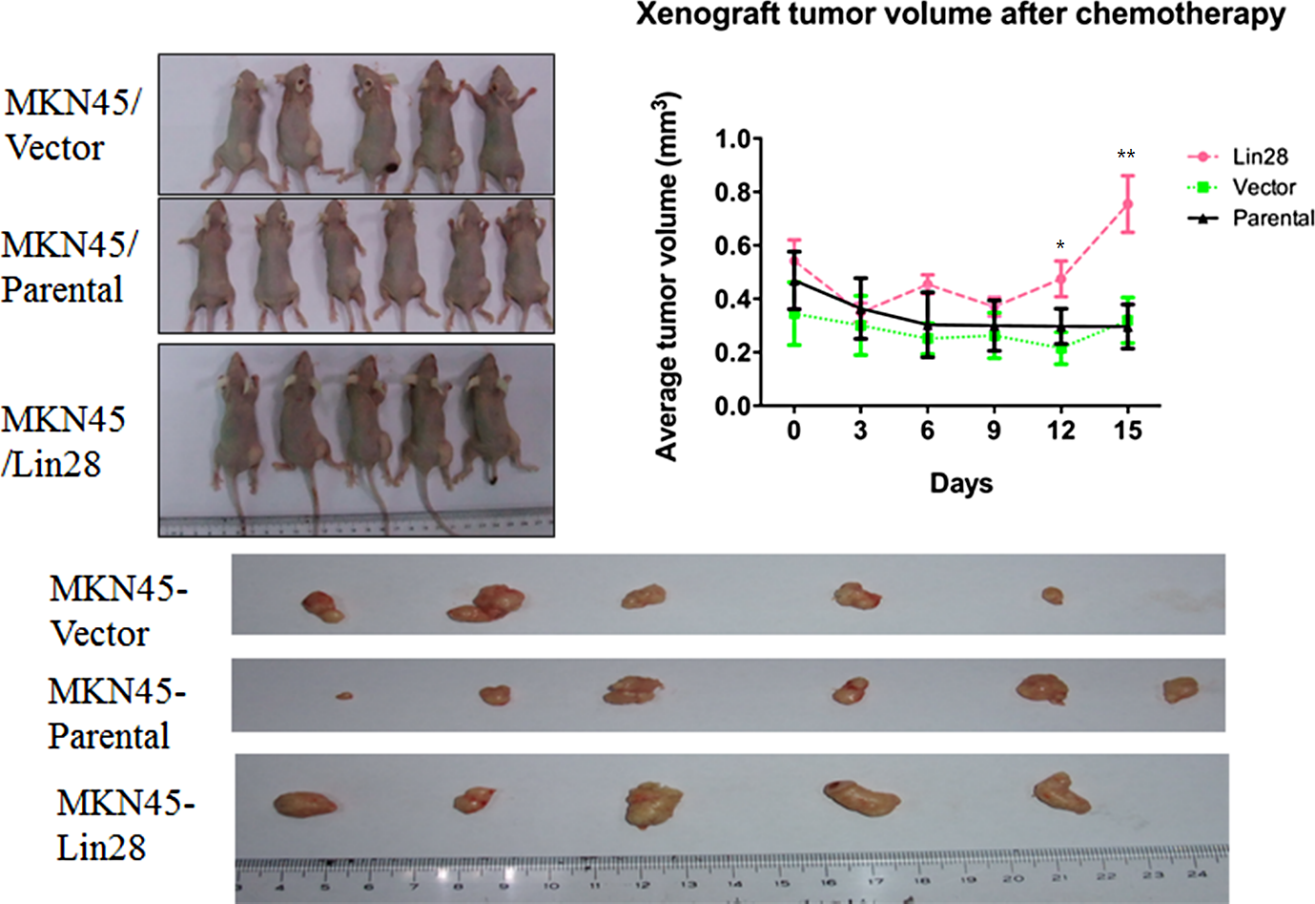
Lin28 increased chemo-resistance of MKN45 *in vivo*. MKN45/Lin28,MKN45/Vector,MKN45/parental cells were inoculated subcutaneously into the axilla of male BALB/c-nu/nu mice. Tumor volume was measured and growth curve was constructed after injection of ADM and OXA *via* tail vein. Each data point represents the mean±SD of three independent experiments. (**p < 0.01).

After 2 cycles of chemotherapy, we found that the xenograft tumors of MKN45/Lin28 cells did not reduce in volume and became bigger than those of other groups (MKN45/Vector and MKN45). In both the MKN45/Lin28 and MKN45/Vector groups, one of the mice died, so chemotherapy was stopped. The tumors were excised and their volumes analyzed. We found that the MKN45/Lin28 xenograft tumors were bigger than the other tumors after ADM and OXA chemotherapy (p<0.01) (Fig 6).

## Discussion

In the present study, we investigated the association of Lin28 expression with chemo-sensitivity in gastric cancer cells. We found that transfection with Lin28 reduces the sensitivity of the gastric cancer cell lines MKN45 and MKN28 to four types of chemotherapeutic reagents (OXA, PTX, ADM, and 5-Fu). Using siRNA interference technology to knockdown Lin28 expression could reverse chemo-resistance. We also showed that miR-107 could down-regulate Lin28 expression, while is also served as one of the potential target microRNAs that is regulated by Lin28. Overexpression of Lin28 up-regulated C-myc and P-gp, while down-regulated Cyclin D1, this phenomenons could reverse by Lin28 siRNA or transfection pre-miR-107. This might be critical mechanisms by which Lin28 decreased chemosensitivity in gastric cancer cells. To our knowledge, this is the first study that demonstrates Lin28/miR-107 signaling pathway might be involved in the chemo-drug resistance in gastric cancer.

Chemotherapy is one of major treatment for gastric cancer patients, but more than 50% of patients will progress to chemotherapy resistance. The possible mechanisms for chemo-resistance in gastric cancer are currently considered to include the trans-membrane transport of ABC transporters, glutathione S transferase, the activity of DNA topoisomerase, cancer gene p53 mutation and apoptosis related pathways. A recent study found that microRNA plays an important role in gastric cancer chemotherapy drug resistance [15]. In a tumor cell resistance model, it was observed that cancer stem cells also play an important role in resistance to chemotherapy. The C-myc gene is one of the surface markers of cancer stem cells but also an oncogene associated with indefinite proliferation, infinite immortalization, and the promotion of cell division [16]. One study reported that 40% of patients with gastric cancer have C-myc gene overexpression and that higher C-myc expression is associated with worse prognosis [16–18]. Studies have also reported a correlation between the C-myc gene and apoptosis. C-myc expression may lead to decreased tumor cell apoptosis. The cell apoptotic rate and sensitivity to induced factors are dependent on C-myc protein levels [19]. Moreover, C-myc overexpression is associated with radiation and chemotherapy resistance. Furthermore, C-myc can up-regulated P-gp, which can induce effluxing of chemotherapeutic reagents led to chemotherapy resistance [20]. Cell cycle arrest can affect the response of cancer cells to certain chemotherapy drugs (such as PTX), which plays a role in G2/S phase reduced drug sensitivity. The cell cycle protein Cyclin D has three subtypes, D1, D2, and D3, and is a cell cycle initiation factor. Its suppression by transforming growth factor -β1 (TGF-β1) prevents cells from entering the cell cycle such that they remain in a resting state [21]. Cyclin D specifically combines with CDK4/6 to phosphorylate and inactivate retinoblastoma protein (pRb), thus contributing to G1/S transition and the initiation of DNA synthesis [21]. In the current study, we found Lin28 were not relevant with Bcl-2, NF-kB and TOPO II in gastric cancer cells. The partnership between Lin28 and microRNAs has been well established, such as let-7, miR-200c, miR-143 [4,22]. Lin28 overexpression down-regulates microRNA, while microRNA can also negatively affect the expression of Lin28. Lin28 together with TUT4 constitutes a negative regulator, which influences maturation and homeostasis of microRNAs [4,22]. By regulating downstream genes, microRNA is involved in cell differentiation and proliferation, cell metabolism, stress responses and the formation of blood vessels. Cheng et al [23] found that after inhibiting miR-107, the growth of HeLa cervical cancer cells and A549 lung cancer cells was promoted. Marijn et al [24] found that the expression of miR-107 in drug-resistant ovarian cancer cells was down-regulated. Our study found that Lin28 can also restrain the maturation of miR-107, and introduction of miR-107 increase gastric cancer cell chemo-sensitivity to OXA, PTX, and ADM. After transfection of anti-miR-107, this sensitivity decreased. Pre-miR-107 caused a decrease in the expression of Lin28, illustrating that the Lin28/miR-107 pathway may be involved in the chemosensitivity changes of gastric cancer.

In summary, we showed that Lin28 could inhibit the expression of miR-107, thereby up-regulating C-myc, P-gp and down-regulating Cyclin D1, subsequently result in chemo-resistance of gastric cancer cells. The Lin28/miR-107 pathway might be served as one of many signaling pathways that is associated with gastric cancer chemo-resistance. Our study may provide new insights into the gastric cancer chemoresistance mechanism. Lin28/miR-107 may be a new potential therapeutic targets for chemotherapy-resistant gastric cancer.

## Supporting Information

S1 Table Minimal data for all these figures. (XLSX)
